# pH-driven shifts in overall and transcriptionally active denitrifiers control gaseous product stoichiometry in growth experiments with extracted bacteria from soil

**DOI:** 10.3389/fmicb.2015.00961

**Published:** 2015-09-24

**Authors:** Kristof Brenzinger, Peter Dörsch, Gesche Braker

**Affiliations:** ^1^Department of Biogeochemistry, Max Planck Institute for Terrestrial MicrobiologyMarburg, Germany; ^2^Department of Environmental Sciences, Norwegian University of Life SciencesÅs, Norway; ^3^University of KielKiel, Germany

**Keywords:** pH, N_2_O, denitrification, *nos*Z, *nirK*, *nirS*, transcriptionally active, extracted cells

## Abstract

Soil pH is a strong regulator for activity as well as for size and composition of denitrifier communities. Low pH not only lowers overall denitrification rates but also influences denitrification kinetics and gaseous product stoichiometry. N_2_O reductase is particularly sensitive to low pH which seems to impair its activity post-transcriptionally, leading to higher net N_2_O production. Little is known about how complex soil denitrifier communities respond to pH change and whether their ability to maintain denitrification over a wider pH range relies on phenotypic redundancy. In the present study, we followed the abundance and composition of an overall and transcriptionally active denitrifier community extracted from a farmed organic soil in Sweden (pH_*H*2*O*_ = 7.1) when exposed to pH 5.4 and drifting back to pH 6.6. The soil was previously shown to retain much of its functioning (low N_2_O/N_2_ ratios) over a wide pH range, suggesting a high functional versatility of the underlying community. We found that denitrifier community composition, abundance and transcription changed throughout incubation concomitant with pH change in the medium, allowing for complete reduction of nitrate to N_2_ with little accumulation of intermediates. When exposed to pH 5.4, the denitrifier community was able to grow but reduced N_2_O to N_2_ only when near-neutral pH was reestablished by the alkalizing metabolic activity of an acid-tolerant part of the community. The genotypes proliferating under these conditions differed from those dominant in the control experiment run at neutral pH. Denitrifiers of the *nirS*-type appeared to be severely suppressed by low pH and *nirK-*type and *nosZ*-containing denitrifiers showed strongly reduced transcriptional activity and growth, even after restoration of neutral pH. Our study suggests that low pH episodes alter transcriptionally active populations which shape denitrifier communities and determine their gas kinetics.

## Introduction

Soil N_2_O emissions from denitrification depend on environmental conditions that control the rates of denitrification and the N_2_O/N_2_ product ratio. Important soil and chemical factors are oxygen availability (as affected by soil moisture and respiration), temperature, nitrate availability and pH (Wijler and Delwiche, 1954; Nömmik, 1956; Firestone, 1982). Among these factors, soil pH is one of the most crucial ones, because it does not only affect overall denitrification rates, but more importantly seems to directly control the N_2_O/(N_2_O + N_2_) ratio of denitrification, and hence N_2_O emission rates from soils (Šimek and Cooper, 2002; Liu et al., 2010; Bakken et al., 2012). Denitrification rates increase with higher pH, whereas N_2_O/(N_2_O + N_2_) ratios decrease (Wijler and Delwiche, 1954; Nömmik, 1956; Dörsch et al., 2012). Direct inhibition of N_2_O reduction by low pH was demonstrated in laboratory experiments with *Paracoccus denitrificans* (Bergaust et al., 2010) and with soils from a long-term liming experiment in Norway (Liu et al., 2010) and may explain the negative correlation between soil pH and N_2_O emission found in certain field studies (e.g., Weslien et al., 2009; Van den Heuvel et al., 2011).

It is well known that pH also affects the composition and size of denitrifier communities in soil. Acidic soils harbor smaller and less diverse 16S rRNA and denitrification gene pools than neutral soils (Fierer and Jackson, 2006; Čuhel et al., 2010; Braker et al., 2012). Acidity seems to be particularly detrimental to *nirS*-type denitrifiers, resulting in a strong decrease of *nirS*/16S rRNA gene ratios (Čuhel et al., 2010). Whether pH-induced changes in taxonomic denitrifier community composition translate into functional differences is unclear. Several studies have linked potential denitrification rates or kinetics to size and composition of denitrifier communities in soils differing in pH (Cavigelli and Robertson, 2001; Bru et al., 2010; Dandie et al., 2011; Braker et al., 2012), suggesting that pH controls soil denitrification and its product stoichiometry via taxonomic differences. In some cases, the relative abundance of marker genes for N_2_O-reducers (*nosZ*) vs. N_2_O-producers (*nirS, nirK, norB*) explained the (N_2_O)/(N_2_O + N_2_) product ratio (Morales et al., 2010; Philippot et al., 2011; Billings and Tiemann, 2014), but this correlation seems to depend on habitat and environmental conditions (Morales et al., 2010; Philippot et al., 2011; Deslippe et al., 2014). In a recent study, Jones et al. (2014) proposed that soil pH controls the abundance of nitrite reductase genes as well as the abundance of the newly discovered *nos*Z Type II clade in soils with relevance to the soil's ability to reduce N_2_O.

The direct effect of low pH on the transcription of denitrification genes has been studied in pure culture (Bergaust et al., 2010), soils (Liu et al., 2010) and cells extracted from soil (Liu et al., 2014). In general, low pH resulted in low numbers of transcripts encoding nitrite reductases (*nirS* and *nirK*) and N_2_O reductase (*nosZ*) (Bergaust et al., 2010; Liu et al., 2010), but the *nosZ*/*nirK* transcript ratio did not change. Interestingly, transcription of *nirS* seemed to be more suppressed by acidity than of *nirK* (Liu et al., 2010), but it is unclear how this affects N_2_O emissions. The underlying molecular mechanisms for direct pH control on N_2_O emissions are not fully resolved, but post-transcriptional impairment of nitrous oxide reductase (N_2_OR) by pH < 6.1 has been suggested (Liu et al., 2014).

Together, this raises three basic questions: (i) is the ability of a soil denitrifier community to reduce N_2_O to N_2_ entirely controlled by pH-impairment of N_2_OR? (ii) do communities harbor organisms which can thrive over a wider pH range without losing N_2_O reductase activity? or (iii) are communities functionally redundant in that they contain distinct members with similar phenotypes adapted to different pH? In the present study, we approached these questions in a model community obtained by extracting microbial cells from a soil with neutral pH. The extracted cells were incubated in pH adjusted batch experiments and we followed the dynamics of denitrifying communities through the analysis of functional genes *nirK, nirS*, and *nosZ* and their gene expression while monitoring gas kinetics at high resolution. The community was extracted from a farmed organic soil in Sweden (SWE, native pH 7.1) which had been previously found to retain much of its functioning (low N_2_O/N_2_ ratios) in pH manipulation experiments (pH 5.4/7.1) (Dörsch et al., 2012). This finding was attributed to a species-rich denitrifier community, and hence to high functional diversity (Braker et al., 2012). Here, we revisited the pH manipulation experiment of Dörsch et al. (2012) and followed functional gene abundance and diversity of the overall denitrifier community (ODC) and the transcriptionally active denitrifier community (TADC) throughout anoxic growth, covering a transient pH range from 5.4 to 7.1. We hypothesized that the inherent alkalization ensuing anoxic growth of denitrifiers induces a succession of taxonomically distinct but, in terms of pH adaptation, functionally redundant denitrifier populations, thus supporting complete denitrification to N_2_ over a wide pH range. Since gene expression does not necessarily result in functional enzymes at low pH (e.g., Bergaust et al., 2010), we compared shifts in transcripts to those in DNA over time, hypothesizing that only taxa expressing functional enzymes would propagate in the growing culture. In this way we assessed whether sustained function (here: complete denitrification to N_2_) would be linked to structural changes in the underlying community.

## Materials and methods

### Soil sample

The soil was originally sampled from a Terric Histosol (FAO) in Sweden and has been used in several studies exploring functional characteristics of denitrification (Holtan-Hartwig et al., 2000, 2002; Dörsch and Bakken, 2004; Klemedtsson et al., 2009; Dörsch et al., 2012) and underlying denitrifier communities (Braker et al., 2012). The neutral pH of the organic soil is due to inclusion of lacustrine limestone from a former lake bottom. Detailed soil characteristics are given in Dörsch et al. (2012). By the time of the present study, the soil had been stored moist at 4°C for 15 years.

### Cell extraction and incubation conditions

Cell extraction was performed as described previously (Dörsch et al., 2012) with the following modification: Instead of two portions of 50 g soils, four portions were used to recover a higher total cell number. Pellets with extracted cells were resuspended in a total volume of 75 mL filter-sterilized bi-distilled water and stirred aerobically for 0.5–1 h to inactivate any existing denitrification enzyme prior to inoculation into a He-washed hypoxic mineral medium (0.7 μM O_2_; see below).

The mineral media contained (L^−1^): 200 mg KH_2_PO_4_, 20 mg CaCl_2_, 40 mg MgSO_4_, 3.8 mg Fe-NaEDTA, 0.056 mg LiCl, 0.111 mg CuSO_4_, 0.056 mg SnCl_2_, 0.778 mg MnCl_2_, 0.111 mg NiSO_4_, 0.111 mg Co(NO_3_)_2_, 0.111 mg TiO_2_, 0.056 mg KI, 0.056 mg KBr, 0.1 mg NaMoO_4_. The medium was buffered with 25 mM HEPES (N-2-hydroxyethylpiperazine-N′-2-ethane-sulfonic acid) and was supplemented with 3 mM of the electron acceptor KNO_3_ and 3 mM Na-glutamate as carbon and nitrogen source. The medium had an initial pH of 5.1. Two aliquots of sterile autoclaved medium were adjusted to pH 5.4 and pH 7.1, respectively, by adding 1 N NaOH to the medium. Two sets (15 each) of 120 mL-flasks were filled with 43 ml of medium of either pH 5.4 or pH 7.1, resulting in 30 sample flasks in total. Additional flasks were used as blanks without adding cells extracted from the soil. The serum flasks were crimp sealed with butyl septa and made near-anoxic (~0.7 μM O_2_) by six cycles of evacuation and He-filling using an automated manifold while stirring the suspension with magnetic stirrers at 500 rpm (Molstad et al., 2007).

### Incubation, gas analyses, and sampling

Denitrification activity was measured directly after inoculation with the cells by denitrification product accumulation. Thirty serum flasks, three blanks, three calibration standards, and two flasks for NO^−^_2_ measurements were placed on a submersible magnetic stirring board (Variomag HP 15; H + P Labortechnik GmbH, Oberschleissheim, Germany) in a 15°C water bath. The water bath is an integrated part of an automated incubation system for the quantification of O_2_ consumption and CO_2_, NO, N_2_O and N_2_ production in denitrifying cultures similar to that described by Molstad et al. (2007). After temperature equilibration, excess He was released by piercing the bottles with a syringe without plunger filled with 2 ml bi-destilled water to avoid entry of air. The bottles were inoculated with 2 mL of cell suspension, yielding approximate cell numbers of 2 × 10^9^ cells per flask (4 × 10^7^ mL^−1^). The headspace concentrations of O_2_, CO_2_, NO, N_2_O, and N_2_ were monitored every 5 h as described by Molstad et al. (2007) and Dörsch et al. (2012).

The incubation experiments were terminated after 210 h when NO^−^_3_-N added to flasks was recovered as N_2_-N. After 0, 12, 26, 48, 70, 96, and 206 h, two to three sample flasks of each pH treatment were sacrificed. Cell densities were determined by spectrophotometry (OD_600_) and NO^−^_2_ concentrations were measured by a spectrometer according to the international standard ISO 6777-1984 (E). The remaining suspension was centrifuged at 4°C and 8.400 × g and the cell pellet was immediately frozen in liquid nitrogen and stored at −80°C until further use. At each time point the pH in the supernatant was determined.

### Extraction of nucleic acids

DNA and RNA were extracted from the frozen cell pellets (−80°C) collected at each sampling point. For this, one or two frozen cell pellets were resuspended in 400 μL sterile water (Sigma-Aldrich, Taufkirchen, Germany). Nucleic acids were extracted using a modified SDS-based protocol (Bürgmann et al., 2003; Pratscher et al., 2011). In brief, the cells were disrupted in a FastPrep beat-beating system and nucleic acids were recovered from the supernatant using a phenol/chloroform/isoamyl alcohol extraction. Subsequently the nucleic acids were precipitated with polyethylene glycol (PEG) 6000 solution and redissolved in 100 μL of sterile (0.1 μm filtered) nuclease-free (DNase-, RNase-free) and protease-free bi-distilled water (Sigma-Aldrich). An aliquot of 20 μL was stored at −20°C for further DNA-based molecular analyses. The remaining 80 μL were treated with RNase-free DNase (Qiagen, Hilden, Germany) for removal of DNA. RNA was purified using the RNeasy Mini Kit (Qiagen), precipitated with 96% EtOH and resuspended in 15 μL nuclease-free water (Sigma-Aldrich) to increase the RNA concentration and stored at −80°C. The integrity of the RNA was checked on a 1.5% w/v agarose gel (Biozym Scientific GmbH, Hessisch Oldendorf, Germany) and the concentration was determined by a NanoDrop1000 instrument (Thermo Fisher Scientific, Dreieich, Germany). The RNA was reverse transcribed with random hexamer primers (Roche, Mannheim, Germany) and M-MLV reverse transcriptase (Promega, Mannheim, Germany).

### Analysis of the composition of *nirK, nirS*, and *nosZ* genes and transcripts

The composition of the denitrifier community was determined by terminal restriction fragment length polymorphism (T-RFLP). The nitrite reductase genes *nirK* and *nirS* as well as the nitrous oxide reductase gene *nosZ* were amplified from cDNA and DNA using the primer pairs nirK1F-nirK5R (~516 bp), nirS1F-nirS6R (~890 bp), and Nos661F-Nos1773R (~1131 bp) and conditions described previously (Braker et al., 1998, 2000; Scala and Kerkhof, 1998). Details on primers and procedures are given in Table S1. These primers were chosen to allow for comparison of the results obtained in this study to previous ones (Braker et al., 2012), although different primers to target these genes have been published more recently (e.g., Green et al., 2010; Verbaendert et al., 2014). The forward *nirS* and *nosZ* primer and the reverse *nirK* primer were 5′-6-carboxyfluorescein labeled. The quantity and quality of the PCR product were analyzed by electrophoresis on a 1.5% w/v agarose gel after staining the gel with 3 × GelRed Nucleic Acid Stain (Biotium, Hayward, CA, USA). PCR products of the expected size were recovered from the gel using the DNA Wizard® SV Gel-and-PCR-Clean-up system (Promega). The PCR products of *nirK, nirS* and *nosZ* were digested using the restriction enzymes FastDigest *HaeIII*, FastDigest *MspI*, and FastDigest *HinP1I* (Thermo Fisher Scientific), respectively, following the manufacturer's specifications. The purified fluorescently labeled restriction fragments were separated on an ABI PRISM 3100 Genetic Analyzer sequencer (Applera Deutschland GmbH, Darmstadt, Germany) and the lengths of fluorescently labeled terminal restriction fragments (T-RFs) were determined by comparison with the internal standard using GeneMapper software (Applied Biosystems). Peaks with fluorescence of >1% of the total fluorescence of a sample and >30 bp length were analyzed by aligning fragments to the internal DNA fragment length standard (X-Rhodamine MapMarker® 30–1000 bp; BioVentures, Murfreesboro, TN). Reproducibility of patterns was confirmed for repeated T-RFLP analysis using the same DNA extracts. A difference of less than two base pairs in estimated length between different profiles was the basis for considering fragments identical. Peak heights from different samples were normalized to identical total fluorescence units by an iterative normalization procedure (Dunbar et al., 2001).

### Quantitative analysis of *nirK, nirS*, and *nosZ* genes and transcripts

The abundance of *nirK, nirS*, and *nosZ* genes and transcripts in the sample flasks was determined by qPCR using primers qnirK876-qnirK1040, qCd3af-qR3cd, and nosZ2F-nosZ2R (Henry et al., 2004, 2006; Kandeler et al., 2006). Details on primers and procedures are given in Table S1. The reaction mixture contained 12.5 μL SyberGreen Jump-Start ReadyMix, 0.5 μM of each primer, 3–4.0 mM MgCl2, 1.0 μL template cDNA or DNA and 200 ng BSA mL^−1^ was added. All qPCR assays were performed in an iCycler (Applied Biosystem, Carlsbad CA, USA). Standard curves were obtained using serial 10-fold dilutions of a known amount of plasmid DNA containing the respective fragment of the *nirK-, nirS-, and nosZ-*gene. Negative controls were always run with water instead of cDNA or DNA. PCR efficiencies for all assays were between 80 and 97% with *r*^2^-values between 0.971 and 0.995.

### Statistical analyses

All statistical analyses and graphics were done using R version 3.0.1 (R Development Core Team, 2013). Significant differences of *nirK, nirS, nosZ*, bacterial 16S rRNA gene and transcript abundance as well as the calculated ratios were assessed using ANOVA (*P* < 0.05). All quantitative data were log-transformed prior to analysis to satisfy the assumptions of homoscedasticity and normally distributed residuals. The community composition changes in the overall and transcriptionally active denitrifier community by T-RFLP were analyzed using non-metric multidimensional scaling (NMDS) and overall differences were tested by ANOSIM (*P* < 0.05). Additionally, differences in the composition of transcriptionally active and overall denitrifier communities (ODC) at a given time point were tested by ANOSIM (*P* < 0.05). An ANOSIM R value near +1 means that there is dissimilarity between the groups, while an *R*-value near 0 indicates no significant dissimilarity between the groups (Clarke, 1993). NMDS analyses were performed with the Bray-Curtis similarity index (including presence and relative abundance of T-RF) which iteratively tries to plot the rank order of similarity of communities in a way that community point distances are exactly expressed on a two-dimensional sheet. The reliability of the test was calculated by a stress-value. Stress >0.05 provides an excellent representation in reduced dimensions, >0.1 very good, >0.2 good, and stress >0.3 provides a poor representation. All community composition data were Hellinger-transformed before analysis, in order to reach normal distribution. ANOSIM, ANOVA, and NMDS were done using package vegan version 2.0-5 (Oksanen et al., 2012).

## Results and discussion

### Denitrification kinetics and shifts in abundance and composition of TADC and ODC at native pH 7.1

**Figure 1:**
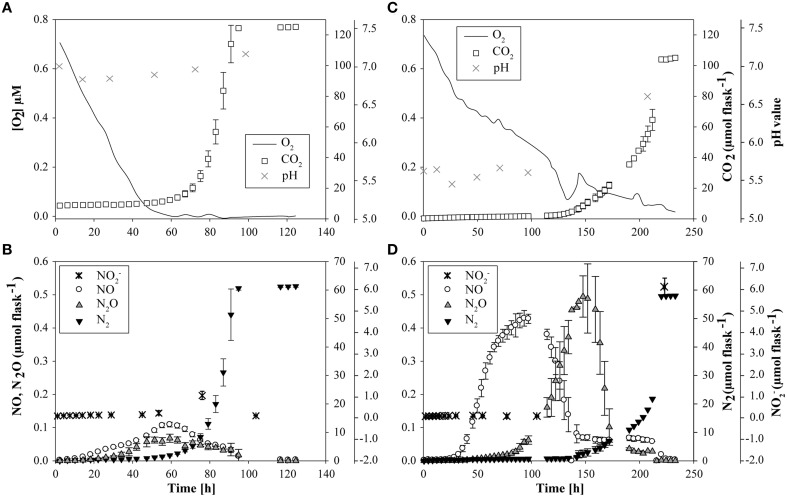
**Accumulation of O_2_, CO_2_, NO, N_2_O, N_2_, NO^−^_2_ and changes in pH value in suspensions of cells extracted from a soil from Sweden at initially pH 7.1 (left panels) and at initially pH 5.4 (right panels) during incubation (0–206 h)**. **(A)** O_2_, CO_2_ concentration and pH value at pH 7.1; **(B)** NO^−^_2_, NO, N_2_O and N_2_ concentration at pH 7.1; **(C)** O_2,_ CO_2_ concentration and pH value at pH 5.4; **(D)** NO^−^_2_, NO, N_2_O and N_2_ concentration at pH 5.4.

**Figure 2:**
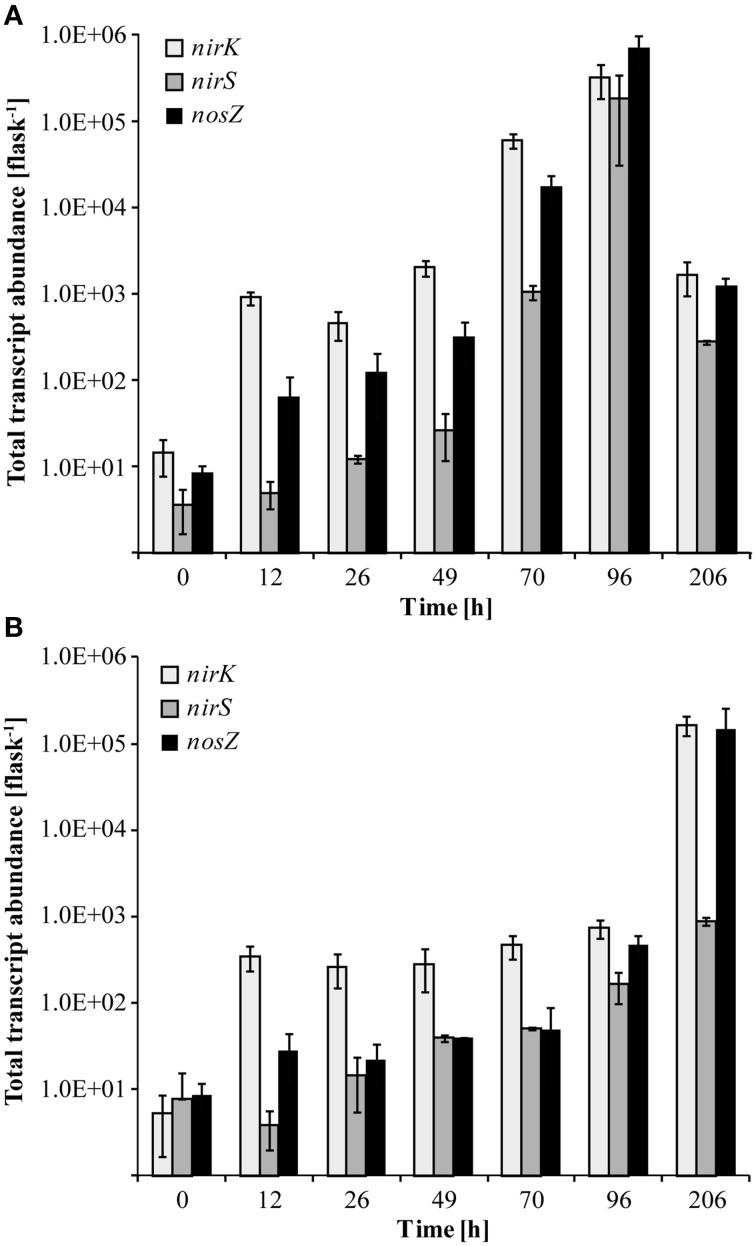
**Transcript abundance of functional marker genes for denitrification (***nirK, nirS***, and ***nosZ***) quantified by qPCR**. **(A)** Transcript copy numbers of the incubation at pH 7.1; **(B)** Transcript copy numbers of the incubation at pH 5.4 (Mean ± SD, *n* = 3).

**Figure 3:**
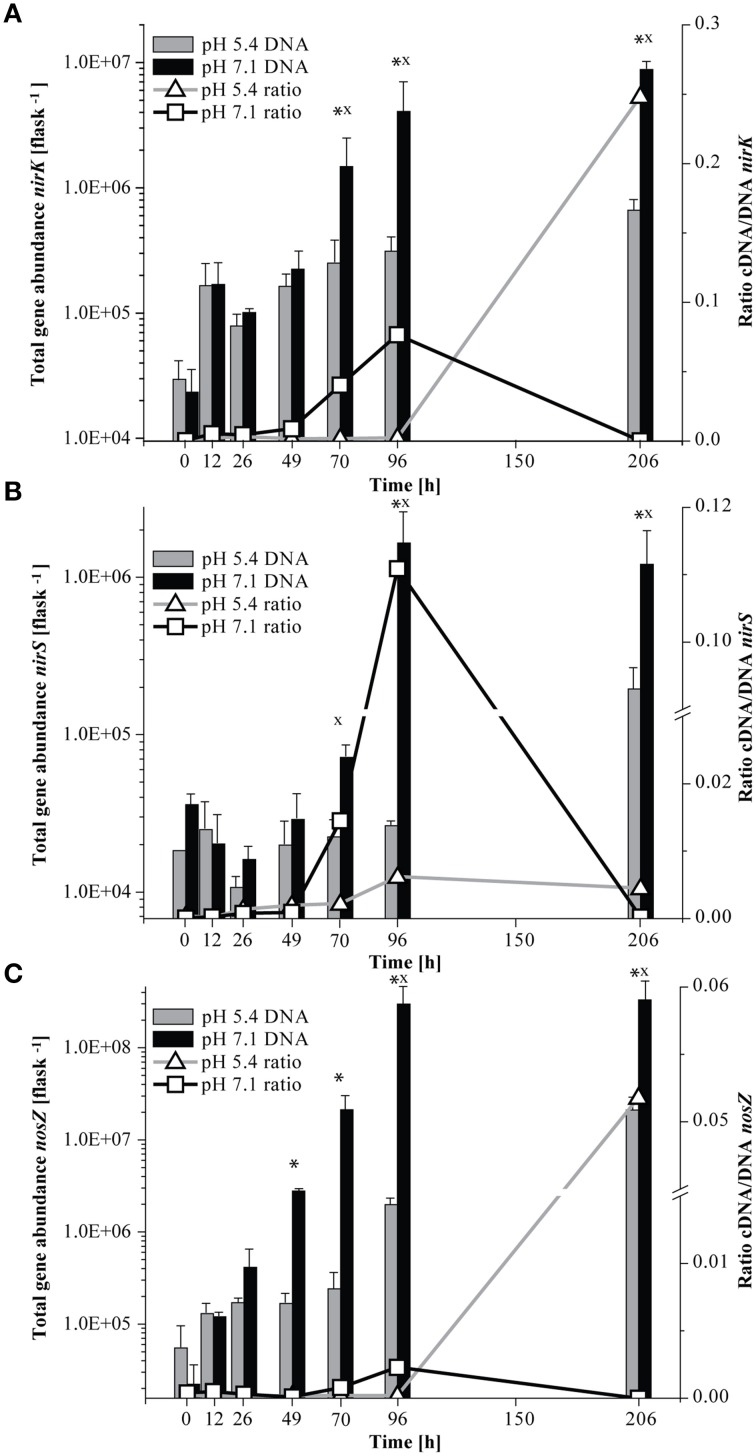
**Abundance of functional marker genes for denitrification (***nirK, nirS***, and ***nosZ***) quantified by qPCR and ratio of cDNA/DNA copy numbers**. Left axis, total gene abundance and right axis, ratio of cDNA/DNA copy numbers. Bars indicate the total gene copy numbers and the line the cDNA/DNA ratio. An asterisk indicates significant differences in gene abundance, x indicates significant differences in the ratio of cDNA/DNA copy numbers between incubation at pH 5.4 and pH 7.1 at a given time point (ANOVA: *P* < 0.05). **(A)** *nirK*; **(B)** *nirS*; **(C)** *nosZ* (Mean±SD, *n* = 3).

**Figure 4:**
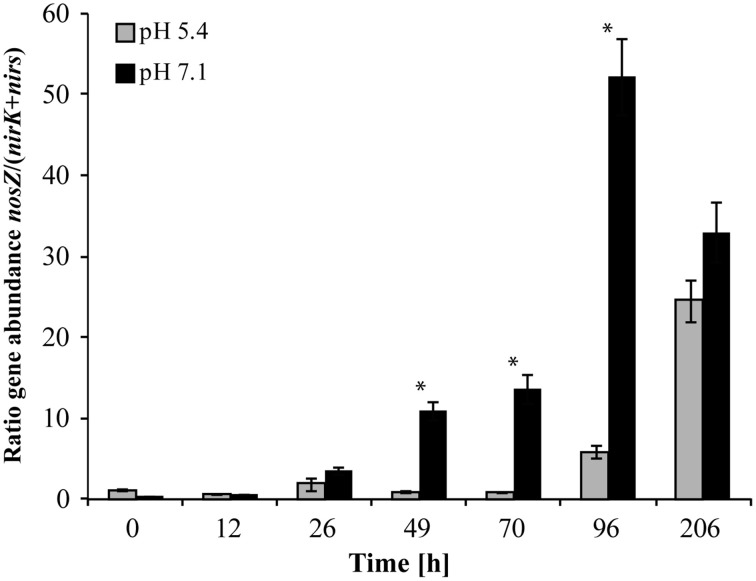
**Ratio of ***nosZ***/(***nirK*** + ***nirS***) gene copy numbers**. An asterisk indicates significant differences in ratios between incubation at pH 5.4 and pH 7.1 at a given time point (ANOVA: *P* < 0.05). (Mean ± SD, *n* = 3).

At native pH 7.1, residual O_2_ after He-washing was depleted and all nitrate was stoichiometrically converted to N_2_ within 96 h of incubation (Figures 1A,B). Net accumulation of gaseous denitrification intermediates was low (< 0.2% of initially present NO^−^_3_-N). Transcriptional activation of functional genes (Figure 2A) and proliferation of denitrifiers containing *nirK* and *nosZ* (Figures 3A,C) started instantly after the cells were transferred to the hypoxic medium. A maximum of relative transcription and community size was reached after 96 h (Figures 3A,C), ~40 h after the start of exponential product accumulation (CO_2_, N_2_) (Figures 1A,B). The maximum relative transcriptional activity (cDNA/DNA ratio) was low with 0.077 for *nirK* (Figure 3A) and 0.002 *nosZ* (Figure 3C), but efficiently translated into denitrifier growth (Figures 3A,C). The strongest growth occurred for *nosZ*-containing denitrifiers (16,500-fold) while denitrifiers of the *nirK*-type grew 400-fold (Table S2). In contrast, growth of *nirS*-type denitrifiers showed a lag-phase of 49 h (Figure 2A, Table S2) after which they were transcriptionally activated (cDNA/DNA ratio of 0.11, Table S3) and increased in abundance, albeit only 50-fold (Figure 3B). Ratios (*nosZ*/[*nirK* + *nirS*]) of >50 after 96 h indicated a tendency of enhanced growth of *nosZ*-type denitrifiers compared to nitrite reducers (Figure 4, Table S4) which may explain the efficient conversion of N_2_O to N_2_ (Philippot et al., 2011). However, PCR-based analyses of genes and transcripts may be biased. The primers used do for instance neither target *nirK* genotypes from *Rhodanobacter* species (Green et al., 2010) nor thermophilic Gram-positive denitrifiers (Verbaendert et al., 2014). The recently postulated *nosZ* clade II (Sanford et al., 2012; Jones et al., 2013) was also not analyzed in this study. Hence, *nosZ*/(*nirK* + *nirS*) ratios and their response to pH must be taken with caution.

**Figure 5:**
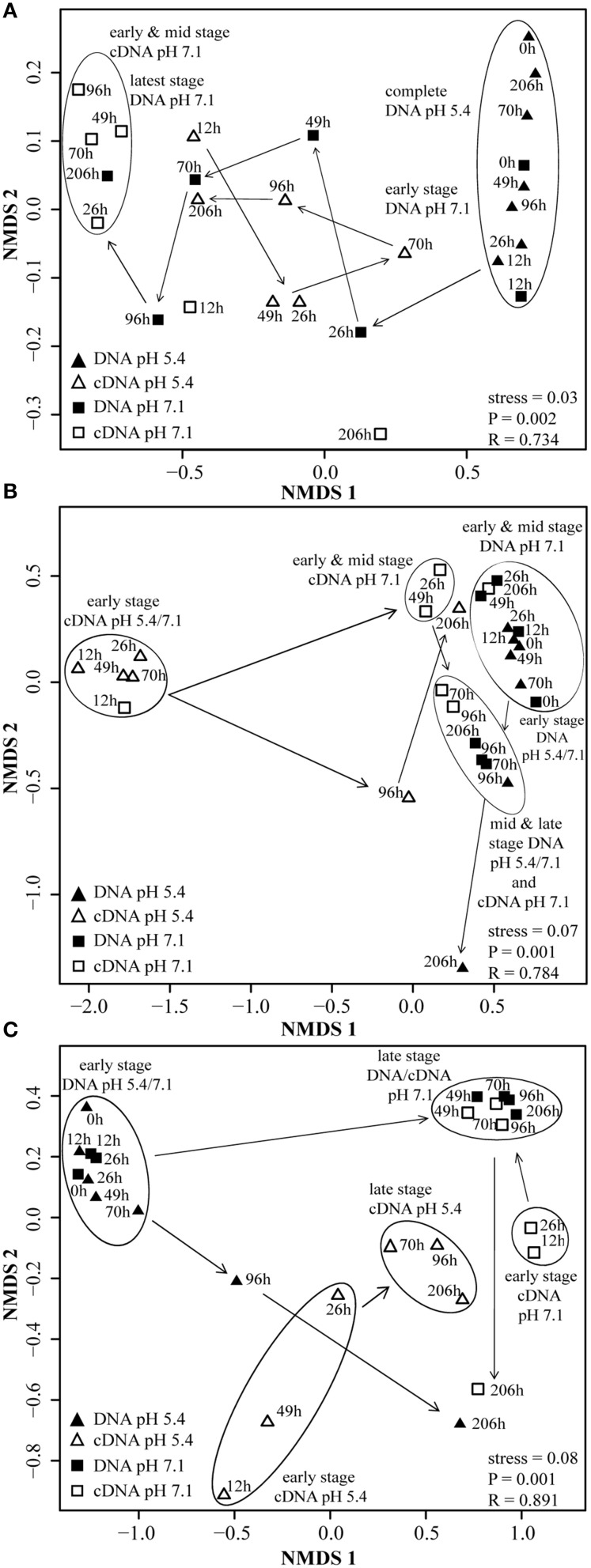
**NMDS plots of denitrifier communities based on cDNA- and DNA-derived T-RFLP analysis of ***nirK, nirS***, and ***nosZ*** from three pooled samples**. Data points represent averaged results of three replicate T-RFLP analyses. Community similarity was calculated by using the statistical program R and the Bray–Curtis similarity measurement, which includes presence and relative abundance of T-RF. Clusters and arrows were inserted manually to highlight clustering and community development. Significant differences in the composition of denitrifier communities at given time points were determined by ANOSIM (*P* < 0.05). **(A)** *nirK*; **(B)** *nirS*; **(C)** *nosZ*.

Community composition data indicated selective transcriptional activity, followed by growth of only a few organisms (Figures S1A, S2A, S3A). Terminal restriction fragments (T-RFs) of 229 bp (representing *nirK* most closely related to *nirK* of *Alcaligenes xylosoxidans*) and of 37 bp length (38 bp *in silico* representing *nosZ* most closely related to *nosZ* of *Pseudomonas denitrificans, Ps. stutzeri*, and *Ps. aeruginosa*), (Table S5) which were of little abundance in or absent from the inocula, respectively, dominated the transcriptionally active *nirK*- and *nosZ*-containing denitrifier communities (Figures S1A, S3A). For *nirS*, a genotype most closely related to *nirS* of *Ps. migulae* (105-bp T-RF) was transcriptionally activated and proliferated that was not even detectable in the initial community (Figure S2A). Still, the composition of the transcriptionally active (TADC) and the overall denitrifier community (ODC) converged throughout the first 96 h of incubation as indicated by multi-dimensional scaling of T-RFs (Figures 5A–C; ANOSIM_26–49*h*_: *P* < 0.05; R between 0.423 and 0.873; ANOSIM_70–96*h*_: *P*>0.05; R between 0.142 and 0.275). The shifts in denitrifier community composition and the decrease in denitrifier diversity (Shannon index, Figures S1A–S3A) did not result in impairment of function, i.e., gaseous intermediates were efficiently taken up and reduced to N_2_ (Figures 1A,B). This suggests that it was not the microbial diversity *per se* that mediated the community's functioning, but the specific metabolic capacities of the dominating denitrifying taxa. Transcription of denitrification genes decreased after all nitrogen oxides were depleted (Figure 2A) and the number of transcripts relative to gene copies became very low (Figures 3A–C). Hence, the increase in diversity and shift in cDNA composition observed for *nirK* and *nosZ*-containing denitrifiers at 206 h was presumably the result of transcript degradation following starvation (Figures S1A, S3A).

### Denitrification kinetics and shifts in abundance and composition of TADC and ODC when exposed to low pH

#### Response of denitrification to incubation at acid pH

Exposing the extracted cells to pH 5.4 showed that most of the functionality in denitrification (low accumulation of denitrification intermediates) was retained (Figure 1D). This was reported earlier for the denitrifying community of this soil (Dörsch et al., 2012). However, denitrification kinetics were clearly influenced by the initially low pH. Respiration activity (measured as CO_2_ accumulation) at pH 5.4 was lower as compared to pH 7.1 (Figure 1C) and NO and N_2_O accumulation started approximately 15 h later (Figure 1D). Net production of NO and N_2_O was four- and nine-fold higher, respectively, than at neutral pH and due to slower denitrification kinetics, the reduction of intermediates occurred sequentially. This is in line with previous studies, finding clear pH effects on the accumulation of intermediates in denitrification (Bergaust et al., 2010; Liu et al., 2010, 2014). For instance, transient accumulation of N_2_O by *P. denitrificans* growing at pH 6.0 was 1500-fold higher than at neutral pH (Bergaust et al., 2010). Liu et al. (2010) found that the production of N_2_ declined to zero with decreasing pH when comparing soils from a long-term liming experiment with *in situ* pH ranging from pH 4.0 to 8.0. Cells extracted from one of the neutral soils and incubated at pH levels between 7.6 and 5.7 for up to 120 h showed a peculiar pH threshold of 6.1, below which no functional N_2_O-reductase was produced (Liu et al., 2014). In our study, nitrate was stoichiometrically converted to N_2_ with less than 1% net N_2_O-N accumulation when incubated at initially pH 5.4 (Figure 1D). However, complete N conversion coincided with a pH shift in the medium (from 5.4 to 6.6) which occurred between 150 and 206 h of incubation (Figures 1C,D). This shift was most likely driven by the strongly increasing denitrification activity during this period. Denitrification is an alkalizing reductive process, consuming 6 moles H^+^ per mol NO^−^_3_ reduced to N_2_. CO_2_ production was clearly coupled to total N-gas production and came to a halt when all N-oxides were reduced to N_2_ (Figure 1C). This suggests that respiratory processes other than denitrification were absent and that the pH-threshold for N_2_O reduction in the medium was overcome by growing denitrifiers which consumed [H^+^] (Figure 1C). This suggestion is further supported by the dominance (>90%) of phylotypes closely related to known denitrifiers at the end of the incubation (Table S6). These findings, together with the transient accumulation of NO at pH 5.4, led us to the conclusion that acid tolerant denitrifiers present in the native community must have been metabolically active at pH 5.4, illustrating the high functional versatility of this community with respect to pH.

#### Response of *nirK* and *nosZ*-containing denitrifier communities to incubation at low pH

We studied how the denitrifier community responded to incubation at initially low pH in terms of growth and transcriptional activation of the denitrification genes *nirK, nirS*, and *nosZ*. Unfortunately, although functional data were collected for the period when the pH shift occurred, due to limitations in the number of samples that could be processed, no community data are available for the period of rapid pH shift. In general, incubation at low pH retarded the transcriptional activation of the functional marker genes (compare Figure 2A and Figure 2B, Table S2). As long as the pH remained stable at about 5.4 (until 96 h), copy numbers of *nirK* and *nosZ* cDNA increased in a range similar to the initial phase of the incubation at pH 7.1 (until 49 h). Moreover, transcriptional activation of *nirK* and *nosZ* at pH 5.4 translated into growth of the communities albeit to a lesser extent than at neutral pH (Figures 3A,C). During the pH shift to 6.6 (96–206 h), presumably concomitant with the exponential accumulation of the N_2_, transcript abundances increased reaching their highest densities at the end of the incubation (Figure 2B). However, the increase in denitrifier density was only 11-fold at most and hence less than at pH 7.1 (Table S2). Hence, although the relative transcriptional activity (ratio of cDNA/DNA copies) of *nirK* and *nosZ* exceeded levels at pH 7.1, transcription seemed not to translate into growth as efficiently.

### Development of transcriptionally active and overall *nirK*-type denitrifier communities when exposed to low pH

Contrary to the incubation at pH 7.1, the composition of the growing ODC in the initially acid incubation changed only marginally and thus differed significantly between the two pH treatments at the end of the experiment. While the development of the ODC at the native pH of the soil (7.1) reflected the composition of the TADC within the first 96 h (see above), this was not the case with initially acidic pH (Figure 5A, Figure S1B). Here, TADC patterns clustered separate (ANOSIM: *P* < 0.05; R between 0.742 and 0.841) from those of the ODC throughout the experiment due to the continuous predominance of the terminal restriction fragment (T-RF) of 229 bp length in the TADC which was of constantly low relative abundance in the ODC (Figure S1B). Thus, we conclude that transcriptional activation of the respective genotypes did not translate into denitrification activity and specific growth of these denitrifiers, suggesting regulation at the post-translational level. Such effects were previously suggested for *nosZ* gene expression in *P. denitrificans* by Bergaust et al. (2010) and confirmed by Liu et al. (2010, 2014) for soils and extracted cells. Bergaust et al. (2010) hypothesized that low pH (6.0) impairs the assembly of N_2_O-reductase in *P. denitrificans*, leading to a dysfunctional enzyme and hence accumulation of N_2_O.

### Development of the transcriptionally active and overall *nosZ*-containing denitrifier communities when exposed to low pH

Incubation at initially pH 5.4 altered the *nosZ-*TADC as well as the *nosZ*-ODC but they remained significantly different (Figure 5C; ANOSIM: *P* < 0.05; R between 0.712 and 0.831). During the first phase of the incubation (up to 70 h) at low pH, growth was small. However, N_2_O-reducers present at very low abundance in the native community seemed to be functional. T-RFLP analysis revealed that after a lag phase of 26 and 70 h, T-RFs of 37 and 40 bp, respectively, that were present at undetectable levels in the ODC, became transcriptionally activated and increased in relative abundance (Figure S3B). After 96 h of incubation, the initial community started to be outcompeted by transcriptionally active *nosZ*-containing organisms. While N_2_O-reducers (40 bp T-RF) were transcriptionally active in the low pH incubation only and started proliferating in the ODC toward the end of the incubation, the T-RF of 37 bp was detected at both pH levels and even dominated the community at neutral pH. Existence of acid-tolerant denitrifiers containing *nosZ* was previously demonstrated for a nutrient poor acidic fen by Palmer et al. (2010) and a riparian ecosystem (Van den Heuvel et al., 2011). Similar to pH 7.1, we observed a tendency of enhanced growth of *nosZ*-containing denitrifiers compared to nitrite reducers as reflected by a *nosZ*/(*nirK* + *nirS*) ratio >25 after 206 h (Figure 4, Table S4) when N_2_O was effectively reduced.

### Transcriptional activity and development of transcriptionally active and overall *nirS*-type denitrifier communities when exposed to low pH

Transcription of *nirS* was not significantly inhibited by low pH and cDNA copy numbers increased slowly until 96 h (Figure 2B). The response in transcription of the community to incubation resembled that during the first 49 h at neutral pH (Figure 2A). When the pH started to shift back to near neutral (pH 6.6) and vigorous proliferation occurred (as judged from N gas kinetics), transcription of *nirS* was further enhanced but the high absolute and relative transcription levels observed for *nirK* and *nosZ* were never reached (Figures 2B, 3B). This contrasts a recently published study with cells extracted from soil (Liu et al., 2014). Liu et al. (2014) observed constantly lower *nirK* and slightly increasing *nirS* and *nosZ* transcript numbers during incubation at pH 5.7 and 6.1, as compared to pH 7.6 where transcripts of all three denitrification genes increased equally. However, in that study, starting conditions were different; the community had a native pH of 6.1 and was preincubated under oxic conditions for several hours. Our findings also contrast other results of Liu et al. (2014), who found stable, pH-independent cDNA/DNA ratios for *nirS* and *nosZ*, whereas for *nirK* the ratio declined due to efficient growth of the *nirK*-type denitrifier community but constant level of transcription at higher pH. We observed persistently reduced relative *nirS* transcription at low pH compared to pH 7.1 and the growth of *nirS*-type denitrifiers was severely inhibited by low pH during the first 96 h of incubation (Figure 3). A previous pure culture study found that already at slightly acidic pH of 6.8, the *nirS*-type denitrifier *P. denitrificans* was unable to build up a functional denitrification pathway (Baumann et al., 1997). Although the nitrite reductase gene was properly induced, the enzyme could not be detected at sufficient amounts in the culture indicating that either translation was inhibited, or once synthesized, nitrite reductase was inactivated, possibly by high concentrations of nitrous acid (HNO_2_). In our study, incubation at low pH did not increase NO^−^_2_ until 96 h (Figure 1D), and accumulation of NO was moderate within the nano-molar range (1 μmol NO in the bottle ~ 730 nM in liquid). Moreover, Baumann et al. (1997) demonstrated that a functional nitrite reductase assembled at pH 7.5 was still active if the culture was shifted to acidic pH. The cells exhibited a reduced overall denitrification activity, but neither nitrite nor any other denitrification intermediate accumulated which is in agreement with our findings (Figure 1D). Despite the low levels of transcription, the *nirS* TADC shifted but only after 96 h of incubation and surprisingly, the ODC changed at the same time, although DNA copy numbers did not increase which cannot be explained. Only with the pH upshift between 96 and 206 h, a slight growth (one order of magnitude) occurred but the community developed distinctly from the TADC (Figure 5B; ANOSIM: *P* < 0.05; R between 0.671 and 0.912). Since the initial abundance of *nirK-* and *nirS*-type denitrifiers in the soil and hence in the inocula was equal, our results indicate a greater robustness of *nirK*-type vs. *nirS*-type denitrifier communities to acidity.

## Concluding discussion

In this study of a model community, we linked transcriptional activation of denitrification genes (*nirK, nirS*, and *nosZ*) and growth of the communities to conversion of nitrogen oxides to N_2_. We found a pronounced succession of TADC and ODC in batch incubations even at neutral pH, suggesting a strong selective pressure on the extracted community. Exposure to low pH (5.4) resulted in (i) sequential and slightly enhanced transient accumulation of denitrification intermediates (NO, N_2_O), (ii) lower and/or retarded transcriptional activation of denitrification genes, together with selective activation of genotypes represented by certain T-RFs and (iii) impaired translation into functional enzymes, with consequences for growth of denitrifier communities. However, since only < 1% of added N accumulated as N_2_O and NO at low pH, and growth of nitrite- (*nirK*-type) and N_2_O-reducers was observed, we conclude that acid-tolerant denitrifier species maintained the functionality of the community as a whole although full conversion of nitrate to N_2_ required extended incubation periods. Experiments altering soil pH *in situ* or in laboratory experiments have repeatedly confirmed that denitrification rates and denitrifying enzyme activity are lower in acidic than in neutral or slightly alkaline soils (Šimek and Cooper, 2002).

Overall, our results show that different mechanisms may determine the response to low pH of a soil denitrifier community adapted to neutral pH:

- Activity and proliferation of *nirK*- and *nosZ*- but not of *nirS*-containing denitrifiers seemed to drive reduction of nitrogen oxides which in turn increased pH. Albeit not at the transcriptional level, growth of *nirS*-type denitrifiers was severely inhibited at low pH and occurred only during or after pH upshift. Acid pH has been repeatedly shown to impair nitrite and particularly N_2_O reduction in certain denitrifiers (e.g., *P. denitrificans*) (Baumann et al., 1997; Bergaust et al., 2010), in soils (Liu et al., 2014) and in cells extracted from soils (Liu et al., 2010), presumably by impairing the assembly of denitrification enzymes post-transcriptionally (Baumann et al., 1997; Bergaust et al., 2010). Here, we could show that expression of *nirK* in some denitrifiers may be affected as well.
- These effects, however, might be compensated by acid-tolerant or acidophilic denitrifier species able to grow and actively denitrify at low pH. Denitrifiers of the *nirK*-type present in the native community of the soil seemed to tolerate a broad range of pH levels as the composition of the growing community remained unaltered during the incubation at low pH.
- Low pH prompted growth of *nosZ*-containing denitrifiers of minor abundance in the native community that were acid-tolerant or even acidophilic. At low pH these *nosZ*-containing denitrifiers seem capable of functionally substituting N_2_O-reducers that were more prevalent in the native community. This agrees well with the functional redundancy hypothesis that distinct species perform similar roles in communities and ecosystems at different environmental conditions, and may therefore be substitutable with little impact on ecosystem processes (Rosenfeld, 2002).

Previous studies have shown that pH-dependent responses in denitrification product ratios in soils were related to the size and composition of the underlying denitrifier communities (Čuhel et al., 2010; Braker et al., 2012). Large variations have been found in the specific activity of e.g., nitrite reductases (50-fold) even between strains of the same species (Ka et al., 1997). The higher susceptibility of *nirS*-type denitrifiers to environmental stressors (e.g., low pH, low C-content) has been repeatedly reported in other studies (Bárta et al., 2010; Čuhel et al., 2010; He et al., 2010). The abundance of *nirS* was also most strongly affected when the pH of a grassland was lowered experimentally for about one year resulting in a high *nosZ*/*nirS* ratio while the *nosZ*/*nirK* ratio remained unaffected (Čuhel et al., 2010). Hence, long-term exposure to low pH in the natural environment will shape soil microbial communities and predetermine a dominance of either *nirK* or *nirS* (Chen et al., 2015). This strongly suggests that taxonomic composition matters for the capability of a soil denitrifier community to effectively denitrify. On the other hand, bulk soil pH is unlikely to be homogeneous in structured soils, probably providing a range of pH habitats distributed throughout the soil matrix. Thus, the occurrence of e.g., N_2_O reduction in acidic soils can be explained by denitrification activity in neutral microsites as proposed by Liu et al. (2014) or by acid-tolerant denitrifiers being present in neutral soils. Consequently, soil denitrifier communities might be comprised of taxa differing in pH sensitivity, which jointly emulate the kinetic response of a soil to pH change.

### Conflict of interest statement

The authors declare that the research was conducted in the absence of any commercial or financial relationships that could be construed as a potential conflict of interest.
