# Supplementary material for: pH-driven shifts in overall and transcriptionally active denitrifiers control gaseous product stoichiometry in growth experiments with extracted bacteria from soil
Source: Front Microbiol. 2015 Sep 24;6:961. doi: 10.3389/fmicb.2015.00961 (PMC4585170; doi:10.3389/fmicb.2015.00961)
Supplement: Supplementary file 1 [file Table1.DOCX]

**Supplementary Table S1.** Primer sets and PCR conditions used to amplify *nirK*, *nirS* and *nosZ* for T-RFLP analysis and cloning (top) and qPCR (bottom).

| **Gene** | **Primer sets** | **Forward primer/Reverse primer** | **Amplicon length (bp)** | **PCR conditions** | **References** |
| --- | --- | --- | --- | --- | --- |
| *nirK* | nirK1F/  nirK5R-FAM | GG(A/C)ATGGT(G/T)CC(C/G)TGGCA/ GCCTCGATCAG(A/G)TT(A/G)TGG | 514 | 95 °C/5min, 10 cycles of (95°C/30sec, 56°C/40sec  (-0.5°C every cycle), 72°C/40sec), 25 cycles (95°C/30sec, 54°C/40sec, 72°C/40sec), 72°C/7min. | Braker *et al*., 1998 |
| *nirS* | nirS1F-FAM/ nirS6R | CCTA(C/T)TGGCCGCC(A/G)CA(A/G)T/ CGTTGAACTT(A/G)CCGGT | 890 | 95 °C/5min, 10 cycles of (95°C/30sec, 56°C/40sec  (-0.5°C every cycle), 72°C/40sec), 25 cycles (95°C/30sec, 54°C/40sec, 72°C/40sec) 72°C/7min. | Braker *et al*., 1998 |
| *nosZ* | nosZ661F-FAM/ nosZ1773R | CGGCTGGGGGCTGACCAA/ ATRTCGATCARCTGBTCGTT | 1100 | 95 °C 5min, 10 cycles of (95°C/30sec, 59°C/90sec  (-0.5°C every cycle), 72°C/2min), 25 cycles (95°C/30sec, 56°C/40sec, 72°C/2min) 72°C/10min. | Scala and Kerkhof, 1998 |

| *nirK* | qnirK876/  qnirK1040 | AT(C/T)GGCGG(A/C/G)A(C/T)GGCGA/  GCCTCGATCAG(A/G)TT(A/G)TGGTT | 165 | 95 °C/15min, 6 cycles of (95°C/15sec, 63°C/30sec (-1°C every cycle), 72°C/30sec, 80°C/15sec), 40 cycles (95°C/15sec, 58°C/30sec, 72°C/30sec, 80°C/15sec), 60 to 95°C (+0.2°C/sec) for denaturation curve. | Henry *et al*., 2004 |
| --- | --- | --- | --- | --- | --- |
| *nirS* | qCd3af/  qR3cd | AACG(C/T)(G/C)AAGGA(A/G)AC(G/C)GG/  GA(G/C)TTCGG(A/G)TG(G/C)GTCTT(G/C)A(C/T)GAA | 425 | 95 °C/15min, 6 cycles of (95°C/15sec, 63°C/30sec (-1°C every cycle), 72°C/30sec, 80°C/15sec), 40 cycles (95°C/15sec, 58°C/30sec, 72°C/30sec, 80°C/15sec), 60 to 95°C (+0.2°C/sec) for denaturation curve. | Kandeler *et al*., 2006 |
| *nosZ* | nosZ2F/  nosZ2R | CGC(A/G)ACGGCAA(G/C)AAGGT(G/C)(A/C)(G/C)(G/C)GT/  CA(G/T)(A/G)TGCA(G/T)(G/C)GC(A/G)TGGCAGAA | 267 | 95 °C/15min, 6 cycles of (95°C/15sec, 65°C/30sec (-1°C every cycle), 72°C/30sec, 80°C/15sec), 40 cycles (95°C/15sec, 60°C/15sec, 72°C/30sec, 80°C/15sec), 60 to 95°C (+0.2°C/sec) for denaturation curve. | Henry *et al*., 2006 |
