# Supplementary material for: pH-driven shifts in overall and transcriptionally active denitrifiers control gaseous product stoichiometry in growth experiments with extracted bacteria from soil
Source: Front Microbiol. 2015 Sep 24;6:961. doi: 10.3389/fmicb.2015.00961 (PMC4585170; doi:10.3389/fmicb.2015.00961)
Supplement: Supplementary file 2 [file Table2.DOCX]

**Supplementary Table S2.** Abundance of functional marker genes for denitrification and of the respective reverse transcribed mRNA (cDNA). Analysis of variance (ANOVA) was performed to test for differences in copy numbers at different sampling times during the incubation at given pH.

| **Time [h]** | ***nirK* DNA**  **pH 7.1** | ***nirK***  **cDNA**  **pH 7.1** | ***nirS* DNA**  **pH 7.1** | ***nirS***  **cDNA**  **pH 7.1** | ***nosZ* DNA**  **pH 7.1** | ***nosZ***  **cDNA**  **pH 7.1** | ***nirK***  **DNA**  **pH 5.4** | ***nirK* cDNA**  **pH 5.4** | ***nirS***  **DNA**  **pH 5.4** | ***nirS* cDNA**  **pH 5.4** | ***nosZ* DNA**  **pH 5.4** | ***nosZ* cDNA**  **pH 5.4** |
| --- | --- | --- | --- | --- | --- | --- | --- | --- | --- | --- | --- | --- |
| 0 | 2.3E+04***^A^***  ± 4.6E+04 | 1.4E+01***^A^***  ± 3.5E+00 | 3.6E+04***^A^***  ± 5.4E+03 | 5.0E+00***^AD^***  ± 8.0E+00 | 2.2E+04***^A^***  ± 1.3E+04 | 8.4E+00***^A^***  ± 3.1E+00 | 3.0E+04***^A^***  ± 1.2E+04 | 5.2E+00***^A^***  ± 2.4E+00 | 1.8E+04***^A^***  ± 9.7E+03 | 7.6E+00***^A^***  ± 8.0E+00 | 4.3E+04***^A^***  ± 3.9E+04 | 8.7E+00***^A^***  ± 2.2E+00 |
| 12 | 1.7E+05***^A^***  ± 6.0E+04 | 9.1E+02***^BCD^***  ± 1.1E+02 | 2.0E+04***^A^***  ± 9.0E+03 | 5.0E+00***^AD^***  ± 1.8E+00 | 1.2E+05***^B^***  ± 1.0E+04 | 6.30E+01***^AB^***  ± 1.5E+01 | 1.6E+05***^BC^***  ± 7.7E+04 | 3.4E+02***^B^***  ± 1.1E+02 | 2.4E+04***^A^***  ± 2.1E+04 | 3.8E+00***^A^***  ± 1.8E+00 | 9.3E+04***^A^***  ± 6.5E+04 | 2.8E+01***^AB^***  ± 1.0E+01 |
| 26 | 1.3E+05***^A^***  ± 4.7E+04 | 4.6E+02***^C^***  ± 1.1E+02 | 1.6E+04***^A^***  ± 3.0E+03 | 9.8E+00***^A^***  ± 9.1E+00 | 4.1E+05***^BC^***  ± 2.2E+05 | 1.2E+02***^B^***  ± 1.1E+01 | 7.9E+04***^AB^***  ± 1.0E+04 | 2.6E+02***^B^***  ± 1.0E+02 | 1.0E+04***^A^***  ± 1.7E+03 | 1.4E+01***^A^***  ± 3.4E+01 | 1.5E+05***^A^***  ± 2.5E+04 | 2.1E+01***^A^***  ± 8.0E+00 |
| 49 | 2.2E+05***^B^***  ± 7.3E+04 | 2.0E+03***^D^***  ± 1.4E+02 | 2.9E+04***^A^***  ± 1.0E+04 | 2.6E+01***^A^***  ± 3.0E+00 | 2.8E+06***^C^***  ± 1.0E+06 | 3.1E+02***^B^***  ± 2.8E+01 | 1.6E+05***^BC^***  ± 3.2E+04 | 2.7E+02***^B^***  ± 1.0E+02 | 1.9E+04***^A^***  ± 7.6E+03 | 3.7E+01***^A^***  ± 2.1E+01 | 1.6E+05***^A^***  ± 4.0E+04 | 4.0E+01***^A^***  ± 2.1E+01 |
| 70 | 1.5E+06***^B^***  ±6.1E+05 | 6.0E+04***^E^***  ± 1.4E+02 | 7.2E+04***^A^***  ± 1.2E+04 | 9.3E+02***^BD^***  ± 1.5E+00 | 2.1E+07***^D^***  ± 8.9E+06 | 1.7E+04***^C^***  ± 2.9E+03 | 2.5E+05***^C^***  ± 1.0E+05 | 4.6E+02***^B^***  ± 1.4E+02 | 2.2E+04***^A^***  ± 5.9E+03 | 5.0E+00***^A^***  ± 1.5E+00 | 2.4E+05***^B^***  ± 1.1E+05 | 4.5E+01***^AB^***  ± 1.0E+01 |
| 96 | 4.1E+06***^B^***  ± 1.3E+06 | 2.6E+05***^F^***  ± 1.7E+02 | 1.7E+06***^B^***  ± 5.6E+05 | 1.4E+05***^C^***  ± 6.6E+01 | 3.0E+08***^E^***  ± 1.8E+08 | 6.8E+05***^D^***  ± 1.0E+05 | 3.1E+05***^C^***  ± 8.4E+04 | 7.1E+02***^B^***  ± 1.2E+02 | 2.6E+04***^A^***  ± 1.3E+03 | 1.6E+02***^B^***  ± 8.8E+01 | 1.9E+06***^B^***  ± 3.0E+05 | 4.6E+02***^B^***  ± 1.4E+02 |
| 206 | 8.8E+06***^B^***  ± 1.0E+06 | 1.7E+03***^BD^***  ± 4.2E+04 | 1.2E+06***^B^***  ± 7.0E+05 | 1.9E+02***^D^***  ± 9.1E+01 | 3.3E+08***^E^***  ± 2.5E+08 | 1.2E+03***^E^***  ± 1.4E+02 | 6.6E+05***^C^***  ± 1.2E+05 | 1.6E+05***^C^***  ± 4.2E+04 | 1.9E+05***^A^***  ± 1.1E+05 | 8.8E+02***^B^***  ± 9.2E+01 | 2.1E+07***^C^***  ± 1.0E+07 | 1.4E+05***^C^***  ± 7.0E+04 |

***^ABCDEF^*** Identical letters behind numbers indicate non-significant differences in copy numbers (*P* < 0.05).
