# Supplementary material for: pH-driven shifts in overall and transcriptionally active denitrifiers control gaseous product stoichiometry in growth experiments with extracted bacteria from soil
Source: Front Microbiol. 2015 Sep 24;6:961. doi: 10.3389/fmicb.2015.00961 (PMC4585170; doi:10.3389/fmicb.2015.00961)
Supplement: Supplementary file 3 [file Table3.DOCX]

**Supplementary Table S3.** Ratios of reverse transcribed mRNA (cDNA) to DNA copies. Analysis of variance (ANOVA) was performed to test for differences in copy numbers at different sampling times during the incubation at given pH.

| **Time [h]** | **Ratio *nirK* cDNA/DNA pH 7.1** | **Ratio *nirS* cDNA/DNA pH 7.1** | **Ratio *nosZ* cDNA/DNA pH 7.1** | **Ratio *nirK* cDNA/DNA pH 5.4** | **Ratio *nirS* cDNA/DNA pH 5.4** | **Ratio *nosZ* cDNA/DNA pH 5.4** |
| --- | --- | --- | --- | --- | --- | --- |
| 0 | 0.0003***^A^***  ± 0.00020 | 0.0001***^A^***  ± 0.00005 | 0.0004***^A^***  ± 0.00013 | 0.0002***^A^***  ± 0.00005 | 0.0004***^A^***  ± 0.00033 | 0.0002***^A^***  ± 0.00010 |
| 12 | 0.0053***^BC^***  ± 0.00295 | 0.0003***^A^***  ± 0.00023 | 0.0005***^AB^***  ± 0.00051 | 0.0021***^B^***  ± 0.00104 | 0.0002***^A^***  ± 0.00020 | 0.0002***^A^***  ± 0.00732 |
| 26 | 0.0046***^B^***  ± 0.00245 | 0.0008***^AB^***  ± 0.00024 | 0.0003***^A^***  ± 0.00006 | 0.0033***^B^***  ± 0.00210 | 0.0014***^A^***  ± 0.00079 | 0.0001***^A^***  ± 0.00003 |
| 49 | 0.0090***^C^***  ± 0.00165 | 0.0009***^AB^***  ± 0.00052 | 0.0001***^A^***  ± 0.00012 | 0.0017***^B^***  ± 0.001053 | 0.0020***^AB^***  ± 0.00137 | 0.0002***^A^***  ± 0.00011 |
| 70 | 0.0403***^C^***  ± 0.03819 | 0.0150***^BC^***  ± 0.0041 | 0.0008***^B^***  ± 0.00106 | 0.0019***^B^***  ± 0.00130 | 0.0022***^A^***  ± 0.0004359 | 0.0002***^A^***  ± 0.00021 |
| 96 | 0.0770***^CD^***  ± 0.04991 | 0.1109***^C^***  ± 0.07318 | 0.0023***^B^***  ± 0.00118 | 0.0026***^B^***  ± 0.00115 | 0.0062***^AB^***  ± 0.00355 | 0.0002***^A^***  ± 0.00009 |
| 206 | 0.0002***^A^***  ± 0.00016 | 0.0002***^A^***  ± 0.00017 | 0.0001***^A^***  ± 0.00005 | 0.2483***^C^***  ± 0.12707 | 0.0045***^A^***  ± 0.03787 | 0.0518***^B^***  ± 0.00334 |

***^ABCDEF^*** Identical letters behind numbers indicate non-significant differences in copy numbers (*P* < 0.05).
