# Supplementary material for: pH-driven shifts in overall and transcriptionally active denitrifiers control gaseous product stoichiometry in growth experiments with extracted bacteria from soil
Source: Front Microbiol. 2015 Sep 24;6:961. doi: 10.3389/fmicb.2015.00961 (PMC4585170; doi:10.3389/fmicb.2015.00961)
Supplement: Supplementary file 4 [file Table4.DOCX]

**Supplementary Table S4.** Ratios of DNA copies of *nosZ/nirK+nirS*. Analysis of variance (ANOVA) was performed to test for differences in copy numbers at different sampling times during the incubation at given pH.

| **Time [h]** | **Ratio *nosZ*/(*nirK*+*nirS*)** | |
| --- | --- | --- |
|  | **pH 7.1** | **pH 5.4** |
| 0 | 0.374***^A^***  ± 0.2351 | 1.147***^A^***  ± 0.5823 |
| 12 | 0.632***^AB^***  ± 0.3080 | 0.683***^A^***  ± 0.1913 |
| 26 | 3.524***^BC^***  ± 2.0967 | 1.910***^AB^***  ± 0.9579 |
| 49 | 11.007***^CD^***  ± 6.0650 | 0.911***^A^***  ± 0.0721 |
| 70 | 13.630***^CDE^***  ± 3.4480 | 0.883***^AB^***  ± 0.7085 |
| 96 | 52.220***^E^***  ± 11.7591 | 5.860***^BC^***  ± 1.4262 |
| 206 | 32.971***^DE^***  ± 4.4718 | 24.573***^C^***  ± 10.7139 |

***^ABCDEF^*** Identical letters behind numbers indicate non-significant differences in copy numbers (*P* < 0.05).
