# Supplementary material for: pH-driven shifts in overall and transcriptionally active denitrifiers control gaseous product stoichiometry in growth experiments with extracted bacteria from soil
Source: Front Microbiol. 2015 Sep 24;6:961. doi: 10.3389/fmicb.2015.00961 (PMC4585170; doi:10.3389/fmicb.2015.00961)
Supplement: Supplementary file 5 [file Table5.DOCX]

**Supplementary Table S5.** Assignment of cloned *nirK*, *nirS*, and *nosZ* gene and cDNA amplicons to terminal restriction fragments. Amplicons of *nirK*, *nirS*, (Braker *et al*., 1998), and *nosZ* (Scala and Kerkhof, 1998) from cDNA and DNA after 206 h of incubation were cloned into the pGEM-T vector and used to transform *Escherichia coli* JM109 competent cells (Promega, Mannheim, Germany). After blue-white selection inserts were sequenced (LGC Genomics, Berlin, Germany) and the nearest neighbor was determined by reconstructing phylogenetic gene trees using the ARB software package (Ludwig *et al*., 2004).

| **Gene** | **T-RF**  **length (bp)** | **pH 5.4** | |  | **pH 7.1** | | **Organisms with most similar gene sequence and respective restriction site** |
| --- | --- | --- | --- | --- | --- | --- | --- |
|  |  | **cDNA** | **DNA** |  | **cDNA** | **DNA** |  |
| *nirK* | 65 | - | + |  | + | + | *Alcaligenes faecalis* |
|  | 106 | + | + |  | - | - | *Pseudomonas entomophila* |
|  | 153 | - | + |  | - | - | *Mesorhizobium* sp. |
|  |  | - | + |  | - | + | *Pseudomonas* sp. G-179 |
|  |  | - | + |  | - | - | *Rhodobacter* sp. |
|  |  | - | + |  | - | - | *Rhodobacter sphaeroides* |
|  | 188 | + | + |  | + | + | *Mesorhizobium* sp. |
|  | 229 | + | + |  | + | + | *Alcaligenes xylosoxidans* |
| *nirS* | 95 | + | + |  | + | + | *Herbaspirillum* sp. |
|  | 99 | - | + |  | + | - | *Pseudomonas fluorescens* |
|  |  | - | + |  | + | + | *Pseudomonas migulea* |
|  | 105 | + | + |  | + | + | *Ps. migulea* |
|  | 141 | + | + |  | - | + | *Ps. migulea* |
| *nosZ* | 38 | - | + |  | - | + | *Brachymonas denitrificans* |
|  |  | + | + |  | + | + | *Pseudomonas stutzeri*,  *Pseudomonas aeruginosa* |
|  | 40 | - | + |  | + | - | *B. denitrificans* |
|  |  | - | + |  | + | - | *Ps. stutzeri*, *Ps. aeruginosa* |
|  | 148 | - | + |  | + | - | *Ps. fluorescens* |
|  |  | + | - |  | - | - | *Ps. stutzeri*, *Ps. aeruginosa* |

bp, base pairs

**+**, sequence with restriction site corresponding to terminal restriction fragment (T-RF)

^-^**,** T-RF not detected
