# Supplementary material for: pH-driven shifts in overall and transcriptionally active denitrifiers control gaseous product stoichiometry in growth experiments with extracted bacteria from soil
Source: Front Microbiol. 2015 Sep 24;6:961. doi: 10.3389/fmicb.2015.00961 (PMC4585170; doi:10.3389/fmicb.2015.00961)
Supplement: Supplementary file 6 [file Table6.DOCX]

**Supplementary Table S6.** Relative abundance of cloned bacterial 16S rRNA cDNA and DNA sequences after 206 h of incubation. Amplicons of 16S rRNA (primers Eub8-27F-Eub1392-1407R; Amann et al., 1995) from cDNA and DNA were cloned into the pGEM-T vector and used to transform *Escherichia coli* JM109 competent cells (Promega). After blue-white selection inserts were sequenced (LGC Genomics) and the nearest neighbor was determined by reconstructing phylogenetic gene trees using the ARB software package (Ludwig et al., 2004).

| **Gene** | **pH 5.4** | |  | **pH 7.1** | | **Organisms with most similar gene sequence and respective restriction site** |
| --- | --- | --- | --- | --- | --- | --- |
|  | **cDNA** | **DNA** |  | **cDNA** | **DNA** |  |
| Bacterial  16S rRNA gene | - | 55% |  | - | 17% | *Pseudomonas frederiksbergensis,*  *Pseudomonas syringae* |
|  | 12% | 29% |  | 4% | 21% | *Ps. fluorescens, Pseudomonas meridiana* |
|  | 12% | - |  | - | - | *Pseudomonas veronii, Ps. fluorescens* |
|  | 10% | 4% |  | - | - | *Pseudomonas tolaasii, Ps. fluorescens* |
|  | - | - |  | - | 13% | *Aquaspirillium arcticum* |
|  | 62% | 4% |  | 96% | 4% | *Herminiimonas glaciei, Herbaspirillum* sp. |
|  | - | - |  | - | 21% | *Paenibacillus wynni, P. borealis* |
|  | - | - |  | - | 8% | *Sphingobacterium* sp. |
|  | 4% | - |  | - |  | *A. xylosoxidans* |
|  | - | 8% |  | - | 16% | others |
