# Supplementary material for: pH-driven shifts in overall and transcriptionally active denitrifiers control gaseous product stoichiometry in growth experiments with extracted bacteria from soil
Source: Front Microbiol. 2015 Sep 24;6:961. doi: 10.3389/fmicb.2015.00961 (PMC4585170; doi:10.3389/fmicb.2015.00961)

**Figure S1.** T-RFLP profiles **(***nirK***)** during incubation at **(A)** pH 7.1 and **(B)** pH 5.4. Left part of a panel, TADC; right part, ODC. Colors of the bars indicate relative abundance of T-RFs. Shannon diversity index is shown above each T-RFLP profile. T-RFs with minimum 1% relative abundance in at least one sample are plotted. (n=3). Numbers in the figure legend indicate lengths of the T-RFs in base pairs.


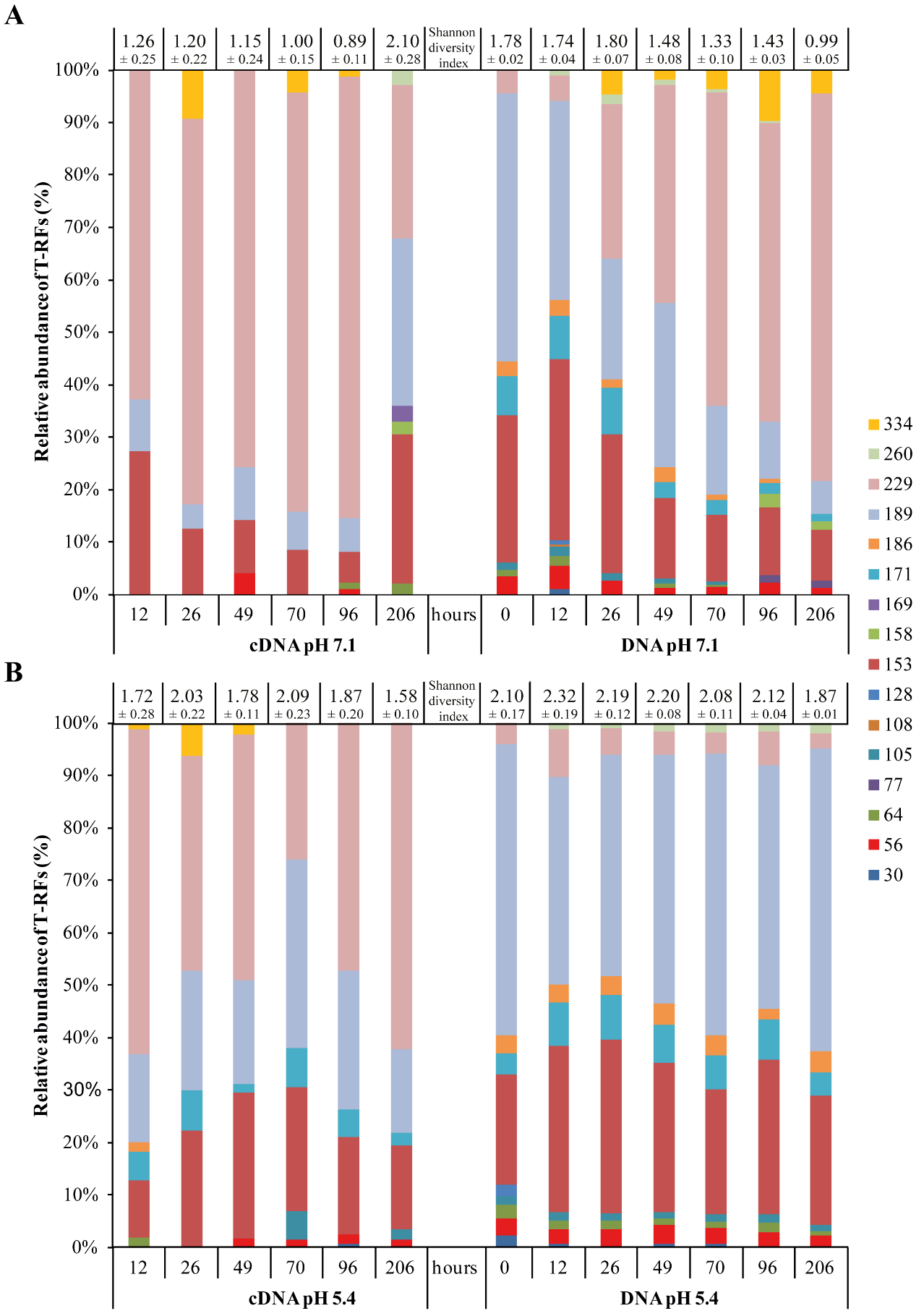


**Figure S2.** T-RFLP profiles **(***nirS***)** during incubation at **(A)** pH 7.1 and **(B)** pH 5.4. Left part of a panel, TADC; right part, ODC. Colors of the bars indicate relative abundance of the T-RFs. Shannon diversity index is shown above each T-RFLP profile. T-RFs with minimum 1% relative abundance in at least one sample are plotted. (n=3). Numbers in the figure legend indicate lengths of the T-RFs in base pairs.


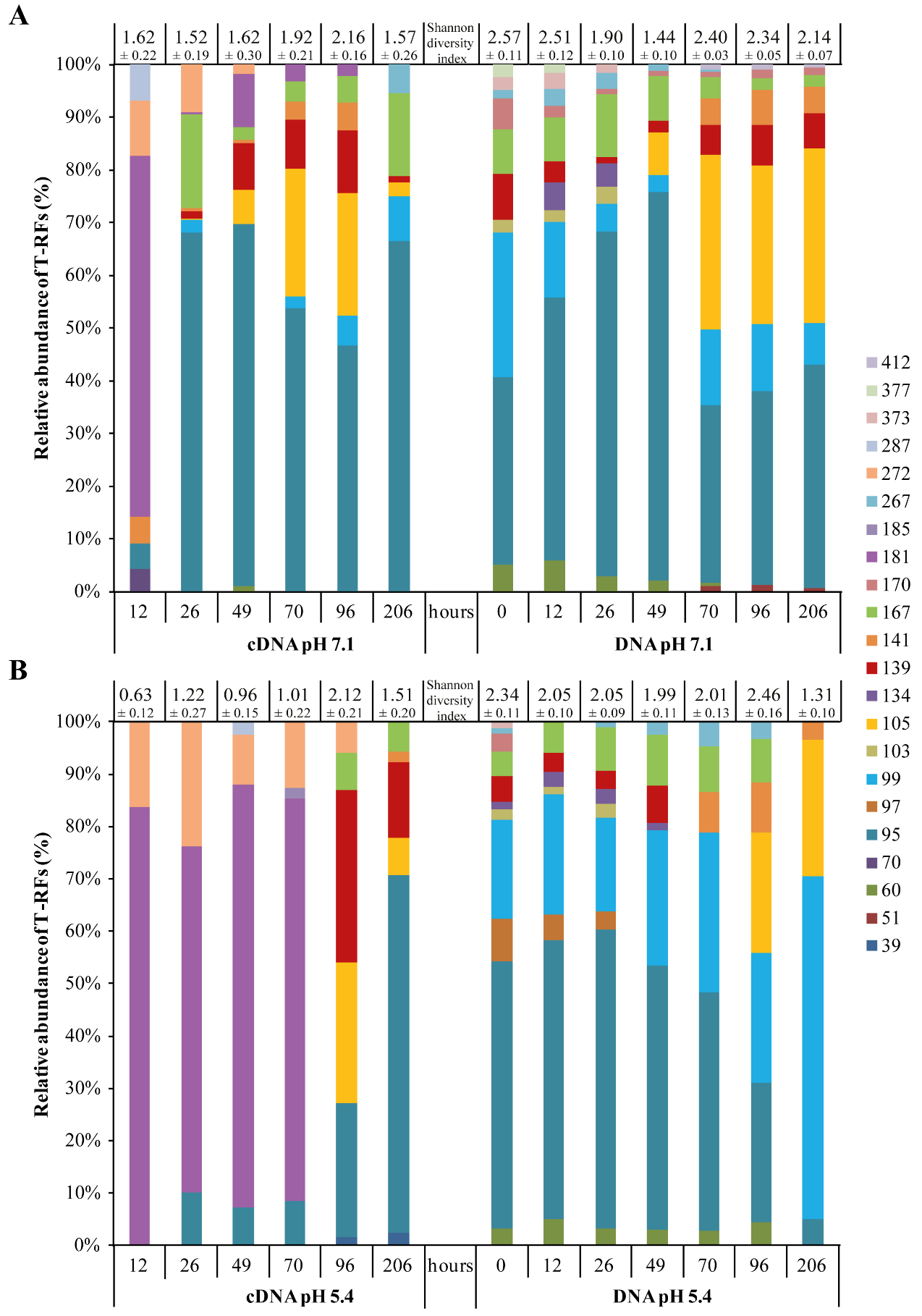


**Figure S3.** T-RFLP profiles **(***nosZ***)** during incubation at **(A)** pH 7.1 and **(B)** pH 5.4. Left part of a panel, TADC; right part, ODC. Colors of the bars indicate relative abundance of the T-RFs Shannon diversity index is shown above each T-RFLP profile. T-RFs with minimum 1% relative abundance in at least one sample are plotted. (n=3). Numbers in the figure legend indicate lengths of the T-RFs in base pairs.


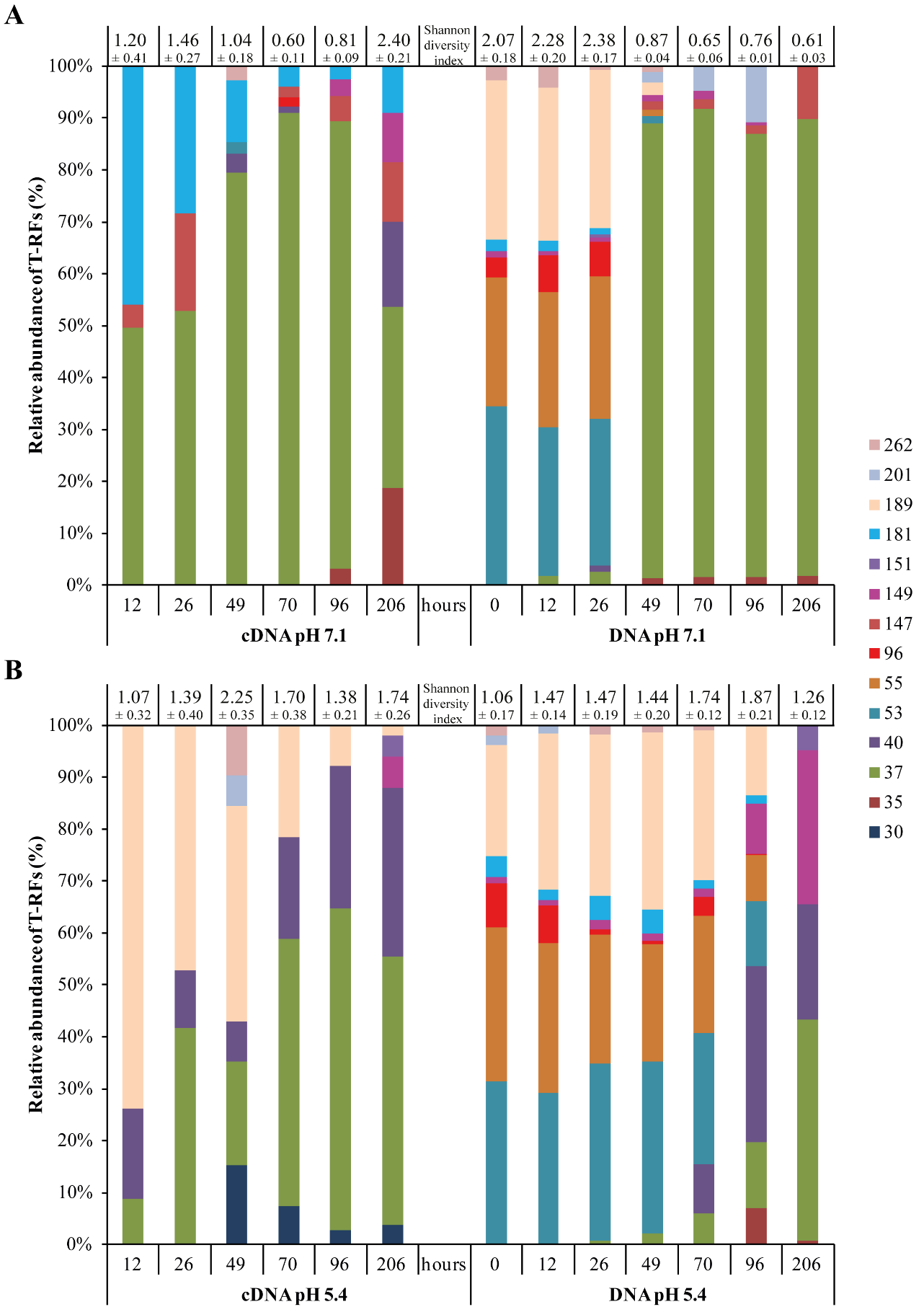

Supplement: Supplementary file 7 [file DataSheet1.DOCX]
